# 73 Green2Gold: Creating and testing ‘Team GB Family Activity Trails’ for improving wellbeing and outdoor physical activity engagement in the East Riding of Yorkshire, England

**DOI:** 10.1093/eurpub/ckae114.073

**Published:** 2024-09-26

**Authors:** Esther Carter, John Saxton, Caroline Douglas

**Affiliations:** University Of Hull, Hull, United Kingdom; University Of Hull, Hull, United Kingdom; University Of Hull, Hull, United Kingdom

## Abstract

**Purpose:**

Systematic review evidence suggests that undertaking green exercise (GE; Coventry et al., 2021) including walking (Hanson & Jones, 2015) can positively impact health and wellbeing. The ‘Green2Gold’ project collaborated with The British Olympic Association to co-create ‘Team GB Family Activity Trails’, a novel GE intervention, in two locations. Signposts were installed along the walking routes containing co-designed activities linked to Olympic sports, physical activity (PA), and nature. Each signpost contained a unique QR code to provide users with additional activities on the project’s website. The study aimed to assess the impact of trail engagement on health, wellbeing, and physical activity.

**Methods:**

In total, 37 participants age 5+ were recruited to test the trails three times within a six-week period. Pre- and post-intervention changes in perceived health, mental wellbeing, and PA and nature engagement were collected and analysed using paired-samples T-tests. Additional post-intervention feedback on enjoyment, motivators, and barriers were collected, with frequencies and crosstabulations calculated. In-the-moment changes in perceived health, mental wellbeing, and enjoyment were measured before and after each walk and compared using two-way repeated measures ANOVAs. Analyses were conducted using JASP v18.1.

**Results:**

Single item scores for general health (p < .001), multiple measures of mental health (p = .002-.043), and number of days engaging in nature (p = .002) significantly improved for both adults and children. Furthermore, significant pre-to-post intervention improvements in perceived value of exercise and being outdoors (p = .015-.031) and the number of days walking per week (p = .034) were reported for adults, with children demonstrating significantly improved future PA intention (p = .043). Overall, a significant effect of ‘Time’ was present for measures of perceived physical and mental health, fitness, nature connection, and inspiration (p < .05) when assessed from pre-to-post walk on three occasions. No significant effect for ‘Walk*Time’ was present.

**Conclusions:**

The study provides preliminary evidence to support ‘Activity Trails’ endorsed by a well-recognised sporting body, such as an Olympic Association, as a free self-directed activity to elicit positive perceived health, mental wellbeing, and nature engagement outcomes at a community level.

**Funding:**

No funding was received for the study.

